# A contribution to the validation of the Italian version of the Body Image Scale (BIS)

**DOI:** 10.1186/s12885-018-5143-6

**Published:** 2018-12-06

**Authors:** Maria Antonietta Annunziata, Barbara Muzzatti, Francesca Bomben, Cristiana Flaiban, Marika Piccinin, Valentina Solfrini

**Affiliations:** Centro di Riferimento Oncologico di Aviano (CRO), IRCCS, 33081 Aviano, Italy

**Keywords:** Body image, Body image scale, Oncology, Psychometrics, Validation

## Abstract

**Background:**

The Body Image Scale (BIS) is a 10-item mono-factorial scale, designed to capture distress and symptoms related to body image in cancer patients. This paper describes the conversion and psychometric evaluation of an Italian BIS version.

**Methods:**

After the back-translation procedure, the Italian version of the BIS, together with the Hospital Anxiety and Depression Scale and the Short Form 36 Health Survey Questionnaire, have been administered to a sample of Italian adult females, surgically treated for a breast cancer at least one year before.

**Results:**

Data on 109 participants were analyzed. The response rate was 92.5%. Response prevalence was adequate for 9 out of 10 items. Principal component analysis showed a one-factor structure. Internal consistency (Cronbach’s alpha =0.924) was good. The BIS correlated with the theoretically pertinent subscales of the other administered tools and was able to discriminate participants (discriminant validity) according to the undertaken surgical treatment (*p* = 0.031).

**Conclusions:**

This study supports the valid and reliable use also of the Italian version of the BIS.

**Electronic supplementary material:**

The online version of this article (10.1186/s12885-018-5143-6) contains supplementary material, which is available to authorized users.

## Background

“Body image” is an individual and composite experience through which people relate to their our body; it depends on many factors including gender, age, physical and mental functioning [1].

The Body Image Scale (BIS, [2]) was developed in England to capture distress and symptoms related to body image in cancer patients. It is a 10-item mono-dimensional scale suitable in oncological settings, regardless of patients’ diagnosis, treatment or disease stages. Respondents answer each item using a 4-point rating scale where Score 0 corresponds to “not at all”, Score 1 corresponds to “a little”, Score 2 corresponds to “quite a bit” and Score 3 corresponds to “very much”. Items’ scores are summed to obtain a total score with higher scores corresponding to higher symptoms/distress. Good psychometric properties were reported in its first validation study [2]. Data on BIS validity and reliability have also been provided for its Portuguese [3], Korean [4], Thai [5], Dutch [6], Spanish [7], and Turkish [8] versions, as well as in studies involving ostomy patients [8], patients undergoing surgery for colorectal cancer [9], or women with benign gynecological conditions [10]. To our knowledge, no studies have been published reporting the validation of BIS for Italian patients [11].

This paper aimed to describe the translation and psychometric validation of an Italian version of the BIS. Our findings will be useful in the Italian national context as it provides an internationally well-known body image assessment tool; at the same time, it will play a role in the in-depth examination of the BIS psychometric properties from a cross-cultural perspective.

## Method

### Participants

Data used for the present study derived from a larger prospective study designed to describe QoL and psychological well-being of breast cancer patients within a month from the cancer diagnosis, and one and two years after it. All participants were adult female breast cancer patients, had to understand the Italian language and signed the informed consent. In addition, a further inclusion criterion for the present study was to have been surgically treated for breast cancer one year before.

### Procedure and materials

BIS was translated into Italian by back translation procedure: two Italian, English proficient, psychology researchers translated the BIS into Italian; then, the two Italian translations were compared and compiled into a single preliminary version; and finally re-translated into English by a professional translator. The final Italian version was achieved by revision of the preliminary one according to the results of the comparison of the original version of the scale and its re-translation into English from Italian.

The final Italian version of BIS is available as an (Additional file 1).

Italian BIS version was administered to participants together with the Hospital Anxiety and Depression scale (HADS, [12]), the Short Form 36 Health Survey Questionnaire (SF-36, [13]), and a form to collect socio-demographic and clinical data.

All participants received materials at home as the second step of a larger study, with the instruction to fill out and return them by mail (a pre-paid envelope was provided together with the study booklet) within 3 weeks.

The Institute Independent Ethics Committee gave its clearance to the study.

HADS is a self-report scale assessing anxious and depressive states of medical patients. It is made up of two factors, in which higher scores correspond to higher anxious and depressive states respectively. Validation data for Italian HADS version were provided by Annunziata et al. [14].

The SF-36 is a QoL measure consisting of 36 items and eight different QoL indices: Physical Functioning, Role-Physical Limitation, Bodily Pain, General Health, Vitality, Social Functioning, Role-Emotional Limitation, Mental Health. In each index, higher scores indicate better functioning in that domain. Validation for SF-36 Italian version was provided by Apolone et al. [15].

Socio-demographic and clinical data were self-reported and collected information on age, marital status, education, occupational status, and cancer treatments.

### Statistical analysis

Feasibility of BIS was assessed by response rates and missing answers.

Response prevalence was defined as the frequency of positive ratings (score > 0) for each item, indicating some change in body image; 30% of positive ratings of the total sample in each item was used as criterion.

To assess the factor structure, a principal component analysis (PCA) was performed. Scree plots, the number of eigenvalues exceeding 1 and the percentage of explained variance were used in determining the number of extracted factors. Only items with factor loadings of 0.40 or above were retained.

Internal consistency was assessed using Cronbach’s Alpha; scores exceeding 0.70 have been considered acceptable [16].

The convergent/divergent validity was assessed by Spearman’s correlations with the subscales of the HADS and SF-36: *rho* < 0.30, 0.30 < *rho* < 0.45, 0.45 < *rho* < 0.60, and *rho* > 0.60 have been considered indices of a negligible, moderate, substantial, and high correlation, respectively [17].

The discriminant validity was assessed comparing BIS score according to the type of received surgery (quadrantectomy vs. mastectomy) through an independent sample t-test.

Descriptive statistics (mean, standard deviation, minimum, maximum) were calculated for the entire scale as well as for each item in this sample.

All analyses were performed on the subsample who had provided a complete BIS; an exception was made for feasibility which was assessed using all provided BIS.

In all analyses, *p* < 0.05 (2-tailed) was used for statistical significance. The Statistical Package for the Social Sciences (SPSS) was used to perform the analyses.

## Results

### Sample characteristics

Study materials were sent to 120 participants of whom, nine (7.5%) did not respond. After exclusion of two participants (one with missing response on BIS and another with two missing responses, 1.7%), 109 participants were included in the dataset. The median age of the final sample was 42 years (range: 26–46). 83.3% participants reported a post-compulsory education (i.e., more than 8 years of schooling); 76.1% reported to be in a stable relationship (i.e., being married or cohabiting); and 80.8% reported having a paying job. 53.2% (*N* = 58) of the sample had received quadrantectomy 1 year before, 42.2% (*N* = 46) mastectomy, and 4.6% (*N* = 5) mastectomy and breast reconstruction.

### Feasibility

The response rate was 92.5%.

There was one (0.9%) missing answer in BIS for item 5, and two (1.8%) missing answers for item 8. No other missing data were present.

### Response prevalence

The frequency of positive ratings for each item ranged from 56% of both Item 3 and 10, to 89% for Item 6; the only exception was item 7, for which the frequency of positive ratings was 27.5%.

### Factor structure

Factor structure was assessed by PCA. Before performing it, data suitability for factoring was verified. Inspection of the correlation matrix revealed the presence of many coefficients of 0.30 and above. Both the Kaiser-Meyer-Oklin value and the Bartlett’s test of sphericity (*p* < 0.001), supported the factorability of the correlation matrix.

PCA revealed the presence of one factors with Eigenvalues exceeding 1, supported by inspection of the scree plot (available as Additional file 2). All items loaded on one component at 0.60 or above, and this solution explained a total of 60.1% of the total variance.

### Internal consistency

Cronbach’s Alpha was equal to 0.924 in the present sample.

### Convergent/divergent validity

Table 1 shows the correlations of the BIS scores with the scores of the HADS and SF-36.

**Table 1:** Spearman’s correlations of BIS – Italian version with HADS and SF-36 *N* = 109)

	BIS	
	Rho	*p*
*HADS*		
Anxiety	0.416	0000
Depression	0.493	0.000
*SF-36*		
Physical functioning	−0.277	0.003
Role-physical limitation	−0.307	0.001
Bodily pain	−0.213	0.026
General health	−0.363	0.000
Vitality	−0.432	0.000
Social functioning	−0.433	0.000
Role-emotional limitation	−0.332	0.000
Mental health	−0.452	0.000

BIS showed a substantial correlation with the subscale Depression of the HADS (positive correlation) and with the subscale Mental Health of SF-36 (negative correlation). In addition, it correlated moderately with the subscale Anxiety of the HADS (positive correlation) and with the SF-36 subscales: Role-Physical Limitation, General Health, Vitality, Social Functioning, Role-Emotional Limitation (negative correlation). Since higher scores in HADS correspond to higher intensity levels in the assessed negative emotional states whereas higher scores in SF-36 correspond to a better functioning in the assessed QoL domain, higher BIS scores correspond to higher levels in anxiety/depression and/or to a poorer QoL.

### Discriminant validity

The subsample of females who had undertaken mastectomy displayed higher BIS scores than who had undertaken quadrantectomy (*M* = 12.94 vs. *M* = 9.78; *p* = 0.031).

### Descriptive statistics

Table 2 displays mean, standard deviation, minimum, and maximum for BIS as a total score and for each BIS item.

**Table 2:** Descriptive statistics for the Italian version of BIS (*N* = 109)

	Mean	Standard deviation	Minimum	Maximum
1. Have you been feeling self-conscious about your appearance?	1.12	0.950	0	3
2. Have you felt less physically attractive as a result of your disease or treatment?	1.38	0.998	0	3
3. Have you been dissatisfied with your appearance when dressed?	0.85	0.901	0	3
4. Have you been feeling less feminine as a result of your disease or treatment?	1.23	1.077	0	3
5. Did you find it difficult to look at yourself naked?	1.25	1.011	0	3
6. Have you been feeling less sexually attractive as a result of your disease or treatment?	1.61	0.932	0	3
7. Did you avoid people because of the way you felt about your appearance?	0.48	0.899	0	3
8. Have you been feeling the treatment has left your body less whole?	1.16	0.925	0	3
9. Have you felt dissatisfied with your body?	1.15	0.970	0	3
10. Have you been dissatisfied with the appearance of your scar?	0.92	1.020	0	3
Total score	11.14	7.472	0	29

## Discussion

Body image is widely recognized as a critical psychosocial issue for cancer patients, with concerns about appearance and body changes varying based upon clinical features of the disease and treatment side effects [18, 19]. Body image concerns can interfere with the disease trajectory and influence both therapeutic decisions and adherence. A negative body image can adversely impact social functioning (intimate, interpersonal relationship), general functioning and QoL during both anticancer treatment and survivorship [19].BIS is a well-known and widely used questionnaire designed to detect distress and suffering related to body image in oncological settings regardless of patients’ diagnosis, treatment or disease stages [11]. Despite its popularity, no data on an Italian version were yet available. The present study described the translation into Italian and the main psychometric properties of a BIS version suitable for Italian cancer patients.

According to the present data, Italian BIS version showed appropriate feasibility, response prevalence, factorial structure, internal consistency, convergent/divergent and discriminant validity. Previous literature [3–8] has already confirmed BIS reproducibility in non-English speaking contexts, our study expanded on this by adding Italy. However, further research is necessary to complete the Italian validation of BIS, as well as to strengthen its the informative power. In fact, more studies are necessary to verify the suitability of BIS with other (than breast cancer women) Italian oncological populations. More in general, BIS ability to discriminate cases from non-cases has not been tested yet in any of the different cultural context (including the original one) in which the tool has been translated. This further step in BIS validation will be useful to improve its clinical relevance. Finally, the present study, together with much of the previous literature on BIS, assessed its factorial structure by means of exploratory methods and, consequently, studies involving confirmatory techniques are recommended.

## Conclusion

In conclusion, the current study confirms the suitability of the BIS to describe body image in Italian female breast cancer patients and represents the first Italian contribution to the validation process for this scale.

## Additional files

Additional file 1: BIS Italian version, BIS, Scala di valutazione dell’immagine corporea. (PDF 124 kb)

Additional file 2: Scree plot. (JPG 16 kb)

## Abbreviations

BIS: Body Image Scale
QoL: Quality of life
