# Supplementary material for: A contribution to the validation of the Italian version of the Body Image Scale (BIS)
Source: BMC Cancer. 2018 Dec 6;18:1222. doi: 10.1186/s12885-018-5143-6 (PMC6282377; doi:10.1186/s12885-018-5143-6)
Supplement: Supplementary file 1 — BIS Italian version, BIS, Scala di valutazione dell’immagine corporea. (PDF 124 kb) [file 12885_2018_5143_MOESM1_ESM.pdf]

A CONTRIBUTION TO THE VALIDATION OF THE ITALIAN VERSION OF THE BODY IMAGE SCALE (BIS)

Maria Antonietta Annunziata,* Barbara Muzzatti,* Francesca Bomben, Cristiana Flaiban, Marika Piccinin, & Valentina Solfrini

Centro di Riferimento Oncologico National Cancer Institute, Aviano (Italy)

BIS – Scala di Valutazione dell'Immagine Corporea

Nel presente questionario Le viene chiesto come si sente relativamente al Suo aspetto e ai cambiamenti che potrebbero essersi verificati a seguito dei trattamenti per la sua malattia. Per favore, legga attentamente ogni domanda e metta una crocetta (**X**) sul punteggio che più si avvicina a ciò che ha provato in quest'ultima settimana.

Nome: ____ Data: _____________

	Per nulla	Poco	Abbastanza	Molto
1. Si è sentita a disagio per il suo aspetto?	0	1	2	3
2. Si è sentita fisicamente meno attraente, in conseguenza della sua malattia o dei trattamenti?	0	1	2	3
3. Si è sentita insoddisfatta del suo aspetto con i vestiti?	0	1	2	3
4. Si è sentita meno femminile in conseguenza della sua malattia o dei trattamenti?	0	1	2	3
5. Si è sentita a disagio nel guardarsi nuda?	0	1	2	3
6. Si è sentita sessualmente meno attraente in conseguenza della malattia o dei trattamenti?	0	1	2	3
7. Ha evitato la gente a causa di come si sentiva per il suo aspetto?	0	1	2	3
8. Ha provato la sensazione che i trattamenti abbiano reso il suo corpo meno integro?	0	1	2	3
9. Si è sentita insoddisfatta del suo corpo?	0	1	2	3
10. E' insoddisfatta per come appare la sua cicatrice?	0	1	2	3
