# Supplementary figures and images for: A contribution to the validation of the Italian version of the Body Image Scale (BIS)
Source: BMC Cancer. 2018 Dec 6;18:1222. doi: 10.1186/s12885-018-5143-6 (PMC6282377; doi:10.1186/s12885-018-5143-6)

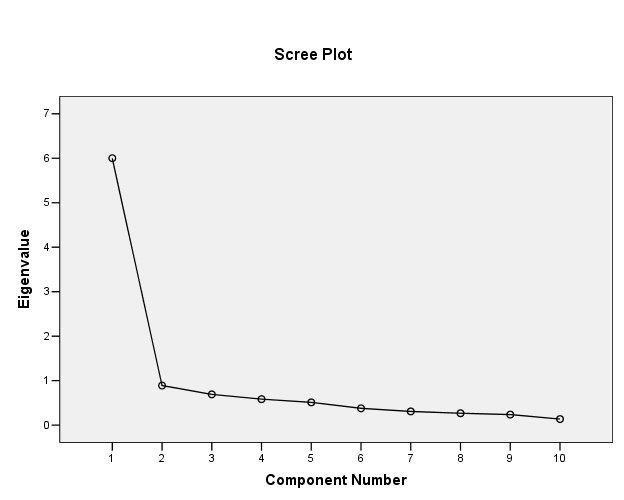

Supplement: Supplementary file 2 — Scree plot. (JPG 16 kb) [file 12885_2018_5143_MOESM2_ESM.jpg]
